# Electron Paramagnetic Resonance Spectra of Pentagonal Bipyramidal Gadolinium Complexes

**DOI:** 10.1021/acs.inorgchem.3c01227

**Published:** 2023-05-12

**Authors:** Jonatan B. Petersen, You-Song Ding, Sandeep Gupta, Aditya Borah, Eric J. L. McInnes, Yan-Zhen Zheng, Ramaswamy Murugavel, Richard E. P. Winpenny

**Affiliations:** † Department of Chemistry, School of Natural Science, The University of Manchester, Oxford Road, Manchester M13 9PL, U.K.; ‡ Frontier Institute of Science and Technology (FIST), Xi’an Jiaotong University, 99 Yanxiang Road, Xi’an 710049, China; § Department of Chemistry, Indian Institute of Technology Bombay, Mumbai 400076, India

## Abstract

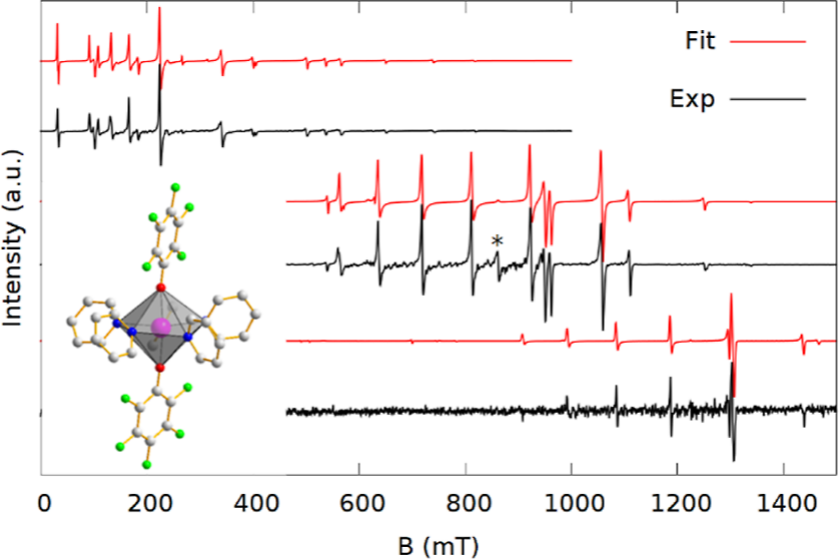

Gadolinium is a special case in spectroscopy because of the near isotropic nature of the 4f^7^ configuration of the +3 oxidation state. Gd^3+^ complexes have been studied in several symmetries to understand the underlying mechanisms of the ground state splitting. The abundance of information in Gd^3+^ spectra can be used as a probe for properties of the other rare earth ions in the same complexes. In this work, the zero-field splitting (ZFS) of a series of Gd^3+^ pentagonal bipyramidal complexes of the form [GdX_1_X_2_(L_eq_)_5_]^*n*+^ [*n* = 1, X = axial ligands: Cl^–^, ^–^O^*t*^Bu, ^–^OArF_5_ or *n* = 3, X = ^*t*^BuPO(NH^*i*^Pr)_2_, L_eq_ = equatorial ligand: Py, THF or H_2_O] with near fivefold symmetry axes along X^1^-Gd-X^2^ was investigated. The ZFS parameters were determined by fitting of room-temperature continuous wave electron paramagnetic resonance (EPR) spectra (at X-, K-, and Q-band) to a spin Hamiltonian incorporating extended Stevens operators compatible with *C*_5_ symmetry. Examination of the acquired parameters led to the conclusion that the ZFS is dominated by the *B*_2_^0^ term and that the magnitude of *B*_2_^0^ is almost entirely dependent on, and inversely proportional to, the donor strength of the axial ligands. Surveying the continuous shape measure and the X^1^-Gd-X^2^ angle of the complexes showed that there is some correlation between the proximity of each complex to *D*_5*h*_ symmetry and the magnitude of the *B*_6_^5^ parameter, but that the deformation of the X^1^-Gd-X^2^ angle is more significant than other distortions. Finally, the magnitude of *B*_2_^0^ was found to be inversely proportional to the thermal barrier for the reversal of the magnetic moment (*U*_eff_) of the corresponding isostructural Dy^3+^ complexes.

## Abstract 2

Multifrequency EPR spectra on a series of seven-coordinate gadolinium(III) complexes doped into yttrium(III) analogues show a well-resolved fine structure. The spectra were simulated with parameters derived from a Hamiltonian based on the presence of a *C*_5_ symmetry axis, but with some additional parameters.

## Introduction

For many years gadolinium(III) has intrigued spectroscopists, with its combination of shielded 4f orbitals and a half-filled shell in the +3 oxidation state and the resulting *L* = 0 ground state with no orbital angular momentum and therefore no first-order spin–orbit coupling.^1^ This results in ground state splitting of typically less than 1 cm^–1^ which is the perfect magnitude for rich EPR spectra as well as relaxation times that are still relatively long.^2^ Gd^3+^ doped into yttrium(III) complexes are presently being studied as potential qubits.^3−5^

Crystal fields determine many of the properties of lanthanide ions and completely dominate their magnetic behavior. For example, the crystal field determines the barrier for reversal of the magnetic moment via the Orbach mechanism in lanthanide single-molecule magnets (SMMs),^6^ and the symmetry of the crystal field is thought to influence the rate of quantum tunneling of the magnetization circumventing this barrier.^7^ We therefore thought it would be worth using the EPR spectroscopy of Gd^3+^ to investigate the crystal field in complexes isostructural with Dy^3+^ SMMs.

Even though it has no formal orbital angular momentum, the splitting of the gadolinium(III) ^8^S_7/2_ ground state still happens through spin–orbit coupling to excited states with *L* ≠ 0 and this splitting adheres to the same symmetry restrictions as crystal field splitting.^8^ It has therefore been suggested that the ZFS parameters obtained for gadolinium can help elucidate how close a family of lanthanide complexes comply with their approximate symmetry.^9^

Most investigations of the lanthanide crystal field and zero-field splitting have been performed in high symmetry environments to ensure the number of parameters needed is low enough to determine a unique best set of parameters from experimental data. In low symmetry, the elucidation of the crystal field parameters often requires ab initio calculations. Methods used such as density functional theory and complete active space self-consistent field (CASSCF) employ approximations that introduce significant errors and for 4f^7^ configurations like Gd^3+^ these errors are on the order of magnitude of the total splitting, rendering theoretical calculations useless for obtaining accurate parameters for the ground state splitting in these systems.^10,11^

Five-fold symmetry does not exist in regular crystals, and it follows that strict fivefold point symmetry is not crystallographically possible. However, molecules with near fivefold symmetry occur. Within the lanthanide series, dysprosium(III) compounds with pentagonal bipyramidal coordination geometries are important as many compounds with this geometry are SMMs with high thermal barriers for loss of magnetization.^12−16^

**Figure 1:**
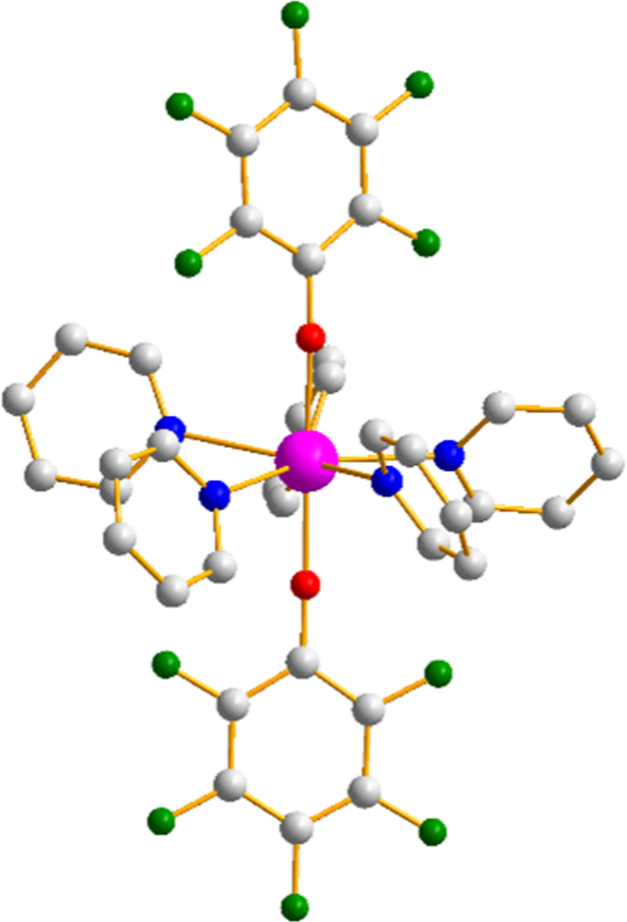
Solid-state structure of [Y(OArF_5_)_2_(Py)_5_]^+^ (**4**) showing the pentagonal bipyramidal coordination geometry. Color code: Y (magenta), F (green), O (red), N (blue), and C (gray). H atoms omitted.

In this work, we set out to investigate five gadolinium complexes with pentagonal bipyramidal coordination geometries (e.g., Figure 1); this geometry has not previously been investigated by EPR spectroscopy. The dysprosium analogues (and in one case even neodymium analogue) are SMMs, which is explained either by the strong axiality of the crystal field or possibly the symmetry.^12−16^ This geometry is ideal for stabilizing the highest *M*_J_ doublet in Dy^3+^ complexes, giving large barriers for reversal of the magnetic moment.^17^ For the current investigation, we measured the room temperature EPR spectra of Gd^3+^ doped into isostructural Y^3+^ complexes at multiple frequencies and examined how well they could be reproduced using a spin Hamiltonian consistent with the approximate symmetry.

## Experimental Section

Six samples were produced for EPR measurements: Gd@[YCl_2_(Py)_5_]BPh_4_·THF **1**; Gd@[YCl_2_(THF)_5_]BPh_4_**2**; Gd@[Y(O^*t*^Bu)Cl(THF)_5_]BPh_4_·2THF **3**; Gd@[Y(OArF_5_)_2_(Py)_5_]B(ArF_5_)_4_·0.5C_6_H_14_**4**; and Gd@[YL_2_(H_2_O)_5_][I]_3_·H_2_O·2L **5a** and [GdL_2_(H_2_O)_5_][I]_3_·H_2_O·2L **5b** [Py = pyridine, THF = tetrahydrofuran, ArF_5_ = pentafluorophenyl, and L = ^*t*^BuPO(NH^*i*^Pr)_2_].

The samples were synthesized by modified versions of the published procedures for the analogous dysprosium complexes with DyX_3_ substituted for YX_3_ and GdX_3_ (X = Cl or I).^13−15,18^ Doping was done by using a mixture of GdCl_3_ and YCl_3_ (1–5% Gd) in the initial synthetic step.

All samples were studied as crystalline powders of yttrium compounds doped with their gadolinium analogue, with the exception of **5b** where the neat gadolinium compound was also measured. Crystallographic parameters are given in Table S1 and relevant geometrical parameters in Table S2.

X-band and Q-band EPR spectra were recorded on a Bruker EMXplus spectrometer equipped with ER 4122 SHQ or ER 5106 QT resonators. K-band EPR spectra were recorded on a Bruker E500 spectrometer equipped with an ER 6706 KT resonator. All spectra were recorded at room temperature with modulation frequencies of 100 kHz and modulation amplitudes of 5–10 G. The recorded spectra were baseline corrected with a first- or second-order polynomial and field corrected against a strong pitch standard sample supplied by Bruker. The samples were measured in sealed quartz tubes as samples **1**–**4** are moisture sensitive.

The spectra were modeled with a spin Hamiltonian of the form
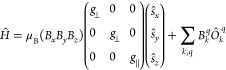
1where μ_B_ is the Bohr magneton. The first term describes the Zeeman interaction between the magnetic moment of the spin (with spin operators , , and ) and the external magnetic field (with components *B*_*x*_, *B*_*y*_, and *B*_*z*_) through an axial *g*-tensor with principal values parallel (*g*_∥_) and perpendicular (*g*_⊥_) to the unique axis (*z*). The second term describes the ZFS of the ground state with Stevens operator equivalents , which are polynomia of spin operators of order *q* < *k*, parameterized with Stevens parameters *B*_*k*_^*q*^.^19^ To adhere to the approximate *C*_5_ symmetry of the complexes, only *B*_2_^0^, *B*_4_^0^, *B*_6_^0^, and *B*_6_^5^ were allowed non-zero values as these are the only allowed operators in the C_5_ point group. Line widths were modeled assuming unresolved hyperfine interactions and strain in the *g*-factors and the dominant ZFS parameter by using axially anisotropic linewidths lw_⊥_ and lw∥ and a Gaussian distribution (strain) around the value of the *B*_2_^0^ parameter.

Spin Hamiltonian parameters were obtained by Levenberg–Marquardt least squares fitting using the multi-purpose EPR software written by Weihe after initial fitting by eye.^20,21^ The parameters were fitted against the spectra of all three frequencies simultaneously, except **5a** and **5b** where each frequency was fit separately to the spectra of both samples simultaneously.

Single crystals of **1**, **2**, and **4** (Y analogues) for crystal structure determination were obtained by recrystallization of the neat compounds from hexane. Single crystals of **5a** and **5b** were obtained directly from the reaction mixture using benzene and dichloromethane as reaction solvents following a similar procedure reported in the literature.^16,18^ X-ray diffraction was measured on Bruker Apex CCD II diffractometer using Mo Kα radiation. Single-crystal X-ray diffraction study for **5b** was performed on a Rigaku Saturn 724+ CCD diffractometer.

## Results and Discussion

**Figure 2:**
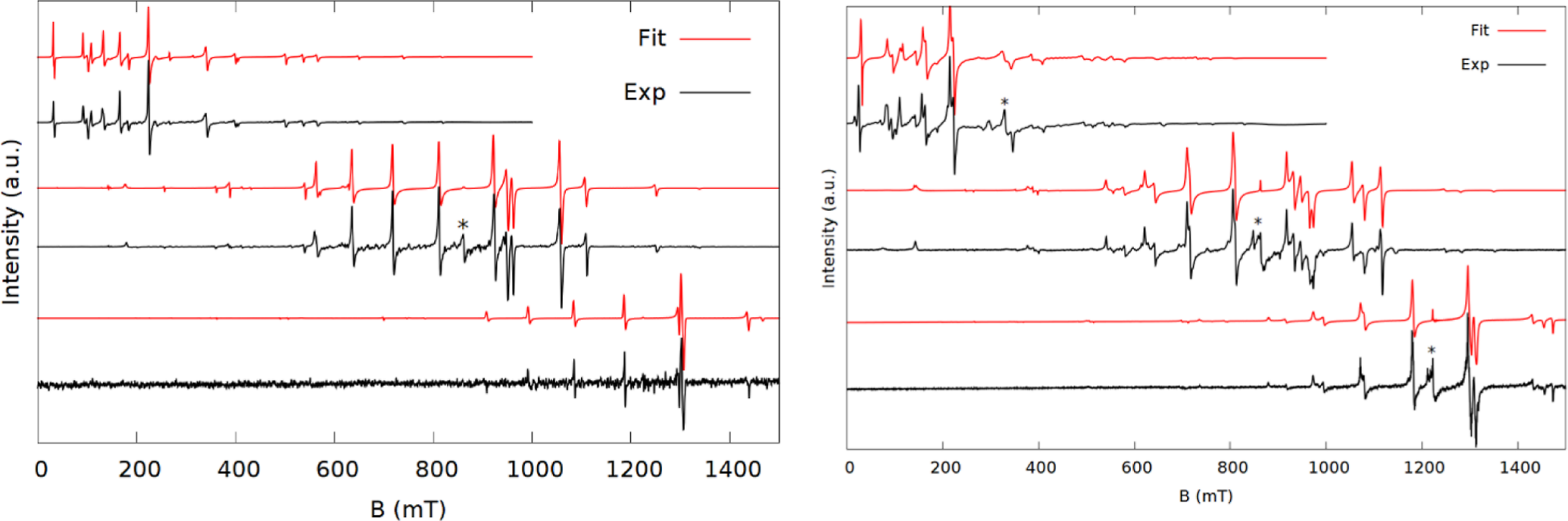
Spectra (black) and simulations (red) at X (top), K (middle), and Q-band (bottom) of **1** (left) and **2** (right). Simulation parameters are based on the parameters in Table 1 and exact frequencies of measurements are given in the Supporting Information. The stars indicate an impurity.

**Figure 3:**
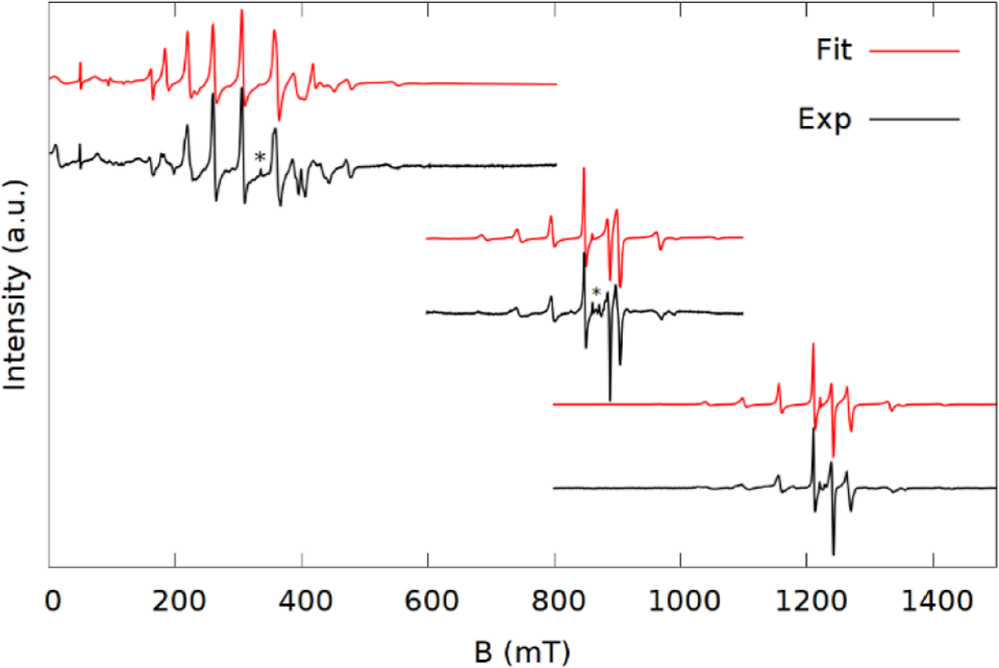
Spectra (black) and simulations (red) of **3** at X (top), K (middle), and Q-band (bottom). The stars indicate an impurity.

**Figure 4:**
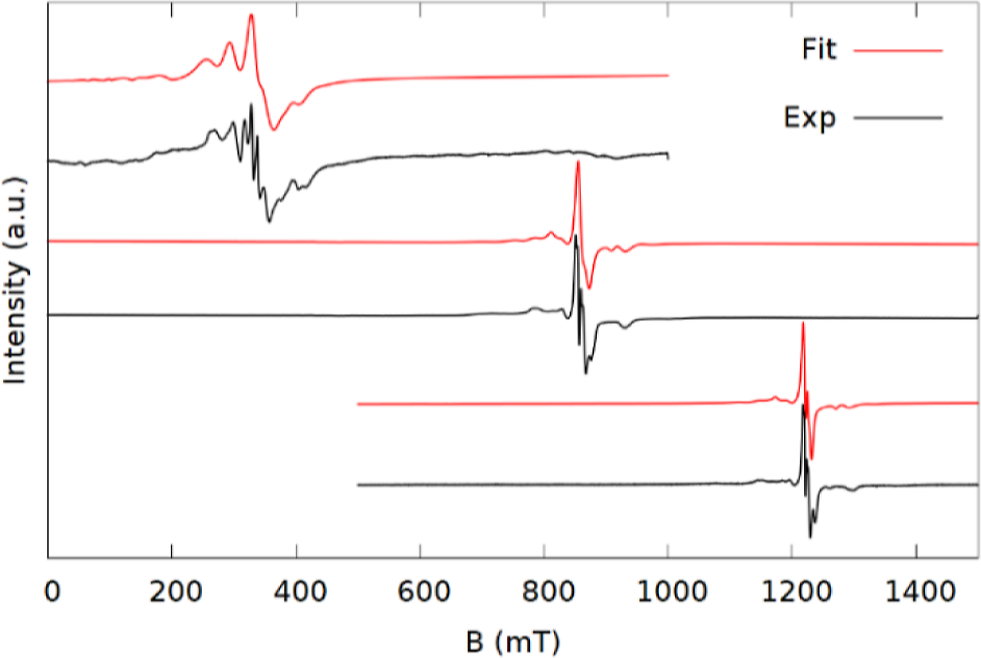
Spectra (black) and simulations (red) of **4** at X (top), K (middle), and Q-band (bottom).

**Figure 5:**
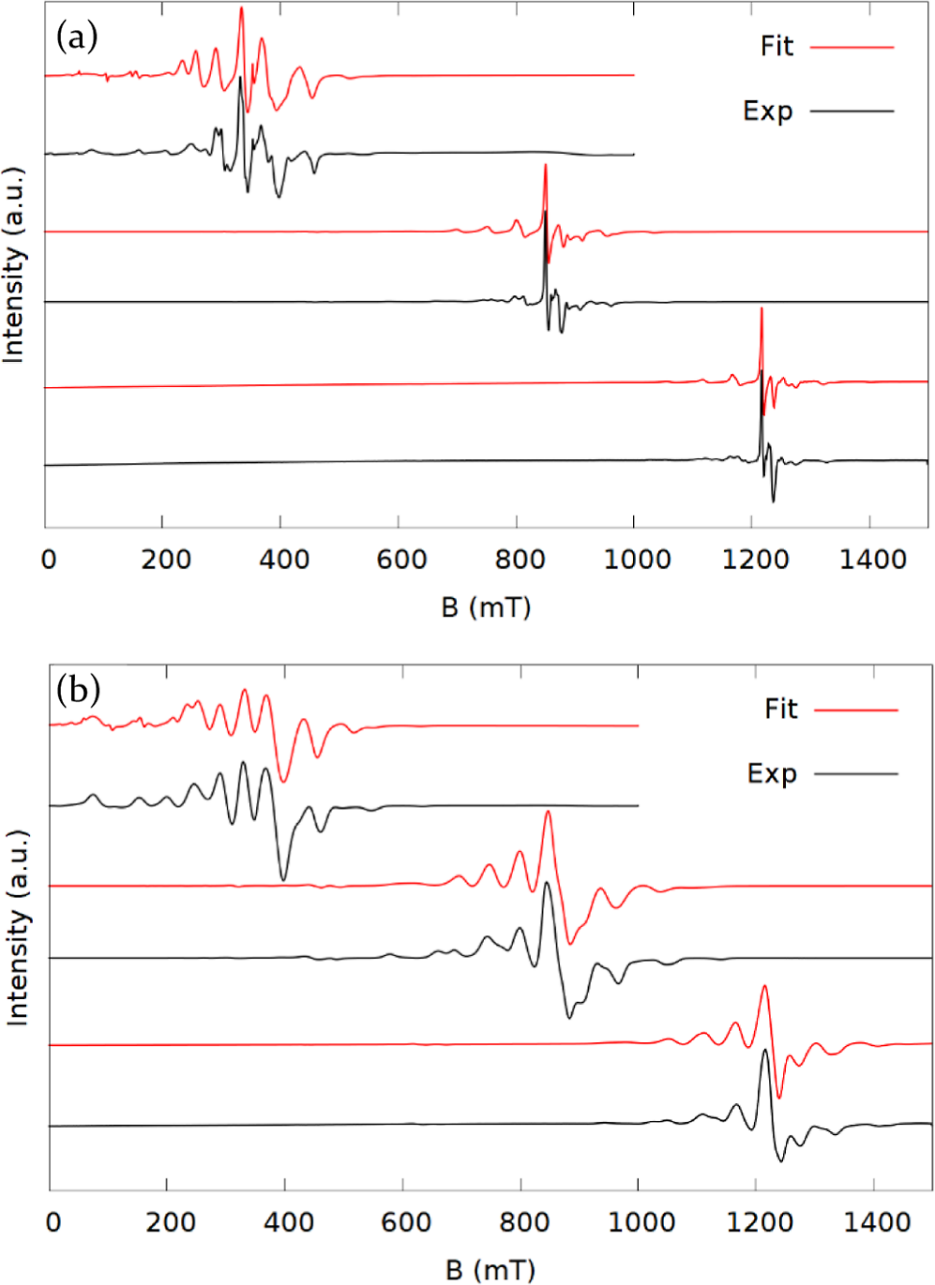
Spectra (black) and simulations (red) at X (top), K (middle), and Q-band (bottom) of **5a** (a) and **5b** (b).

The experimental spectra of **1–5b** are shown along with the best fit simulations in Figures 2–5. All the samples gave intense EPR signals and spectra with many observable transitions. In the K-band spectra, both Δ*m*_s_ = ±1 and Δ*m*_s_ = ±2 (<500 mT) transitions are observed for all samples with the exception of **3** where the smaller sample size meant that the field range was cut short to focus on the main part of the spectrum. The Δ*m*_s_ = ±2 transitions are also accurately reproduced in the simulations, substantiating the validity of the model.

The spectra of **1** and **2** contain several intense narrow peaks and are presented in Figure 2. The simulation parameters used are given in Table 1. Both have the Δ*m*_s_ = ±1 transitions spread over a wide range of as much as 800 mT, indicating large ZFS.

**Table 1:** Best Fit Spin Hamiltonian Parameters for **1–5** from EPR Spectra and Structural Data from Gadolinium Crystal Structures^a^

	*g*_⊥_	*g*_∥_	*B*_2_^0^ (10^–2^cm^–1^)	*B*_4_^0^ (10^–5^cm^–1^)	*B*_6_^0^ (10^–7^cm^–1^)	*B*_6_^5^ (10^–5^cm^–1^)	*B*_2_^2^ (10^–3^cm^–1^)	X_1_-Gd-X_2_ (deg)	CShM *D*_5*h*_	*B*_6_^5^/Δ*E*^f^ (10^–5^)	*U*_eff_^g^ (K)
1	1.994(1)		3.63(1)	–1.3(1)	0	0.6(5)	0	176.8	0.093	0.46	h
2^b^	1.993(1)		3.705(2)	–0.92(4)	0	0	1.83(5)	179.3	0.244	3.7^e^	78
								176.2	0.224		
3	1.992(1)		1.86(2)	–1.6(2)	0	–1.7(4)	0	178.7^c^	0.274^c^	–2.5	950
4	1.997(1)	1.991(2)	1.01(8)	–2(1)	–10(7)	–8(2)	0	178.4^c^	0.979^c^	–22	700
5^d^	1.994(3)	1.999(5)	1.58(8)	–3(1)	–6(5)	–7(2)	0	174.5^c^	0.173^c^	–12	735.4

a Numbers in parentheses are estimated standard deviations of the last digit.

b Crystal structure contains two Gd sites in the asymmetric unit.

c Data from Y analogue crystal structure.

d Spin-Hamiltonian parameters from simultaneous fit of **5a** and **5b** K-band spectra.

e See Table S3 for the fit of **2** with *B*_6_^5^.

f Δ*E* is the total splitting of the ^8^S_7/2_ multiplet (separation of top and bottom Kramers doublet) in zero-field for the parameters in the table.

g *U*_eff_ is the thermal barrier for the reversal of magnetization in the analogous Dy^3+^ complex.

h Not measured.

The multitude of very weak peaks, between the intense peaks, are caused by polycrystallinity effects, meaning that larger crystallites give more weight to certain orientations rather than a true average of the orientations. To prove this, the sample was turned 10° and a new spectrum measured where the position and shape of these minor peaks changed. This effect was seen even though the sample was thoroughly ground, which is due to the extraordinarily narrow linewidths and wide spectral range of **2**.

The spectra of **1** can be fit with the axial Hamiltonian (1) (Figure 2, left). The spectra of **2** are of similar spread, but have more transitions (Figure 2, right) than can be accounted for with axial symmetry. This could be because the asymmetric unit of the crystal structure contains two gadolinium complexes with slightly different geometries. The main peaks are at positions similar to those in the spectra of **1** and the best fit parameters of **1** were used as the starting point for fitting of **2**. The resulting simulated spectra resemble the experimental data, but the fitted parameters are less reliable (Figure S2). An attempt at fitting the spectra with two independent sets of axial parameters failed, as the spectra are too convoluted. We found that on exchanging *B*_6_^5^ for *B*_2_^2^ in the spin Hamiltonian, i.e., decreasing the symmetry, the spectra can be simulated almost to perfection (see Figure 2) and yields very similar values of *B*_2_^0^ and *B*_4_^0^ (Table 1). Deciding which parameter to include from *B*_6_^5^ and *B*_2_^2^ is not immediately obvious but the fit is noticeably better with rhombic symmetry for **2**, i.e., lower than *C*_5_ symmetry. A full single-crystal study EPR would be required for an unambiguous assignment.

In contrast to **1** and **2**, the signals of **3** are found in a much narrower field range, indicating smaller ZFS, though still large enough that all features are resolved. The spectra could be modeled with the same set of axial ZFS terms, but different values, as used for **1**. An attempt was made to fit **3** with the *B*_6_^5^ parameter exchanged with *B*_2_^2^, as was necessary for **2**, but this made no significant improvement. Hence, the ZFS of **3** conforms to the approximate *C*_5_ symmetry.

In some of the spectra of **1**, **2**, and **3** a peak corresponding to a *g*-value of 1.993–1.995 is seen. This peak does not fit with the simulations, and we attribute it to a small impurity of an amorphous Gd species giving rise to an isotropic signal.

The spectra of **4** are less well resolved than for the other compounds. The spectra only extend over roughly 200 mT and, apart from one transition in the middle, the transitions have broader linewidths than the other spectra. The narrow spectral range is a result of a small ZFS of the ground state. The spectra can be simulated with a relatively large *B*_6_^5^ parameter, though to reproduce the linewidths a significant strain of the *B*_2_^0^ parameter with a standard deviation of 7.3% was needed. This is a great deal more than necessary for the other samples (>1%).

**5** was measured both as a doped (**5a**) and neat (**5b**) compound. Both samples give rich spectra (Figure 5) which is surprising as neat gadolinium complexes often have line widths so broad that few transitions are observable. For comparison, Figure S3 shows the spectrum of neat GdCl_2_(THF)_5_, which has linewidths so large that the spectrum resembles a single transition.

The narrow line widths of **5b**, could be the result of the crystal structure containing both water and two additional uncoordinated ligand molecules and their iodide counterions increasing the distance between neighboring molecules and hence a lower density of paramagnetic species. Like **4** and to some degree **3** the transitions of **5a** are narrower toward the middle of the spectrum than at the edges, which again suggests an influential *B*_6_^5^ parameter or strain on *B*_2_^0^. This effect is not seen in the spectrum of **5b**. The spectra of **5a** and **5b** complement each other well, with **5a** having narrow line width on the central transitions, giving a good measure of the *g*-values and **5b** relatively even linewidths and thus giving a better fit of the splitting. The two samples contain the same complex and approximately the same ZFS would be expected, since the ionic radius of Y^3+^ is similar to that of Gd^3+^ (102 and 105 pm, respectively, in eight coordinate complexes).^22^ They were therefore fitted together to give a single set of spin-Hamiltonian parameters.

As fitting six data sets at a time was too cumbersome, the spectra were fitted in pairs of **5a** and **5b** at each frequency, starting with the K-band and then using the resulting parameters as a starting point for X- and Q-bands. The best fit parameters are presented in Table S4. The ZFS parameters obtained this way are mostly consistent, apart from *B*_4_^0^ which is an order of magnitude lower at X-band. The Zeeman parameters give conflicting values. X- and Q-band *g*-values (see Table S4) are lower than those from the K-band spectrum but within experimental error. The three parameter sets have the same trend with *g*_⊥_ < *g*_∥_ by 0.005–0.009.

Overall, the fits match the experimental data well and give the well-defined parameters in Table 1. Since the spectra were recorded at room temperature, simulations are not sensitive to the sign of the ZFS parameters, though they are to their magnitude and relative signs. The parameters in Table 1 are arbitrarily written with positive values of *B*_2_^0^.

Due to the high energy of the excited states of Gd^3+^, its *g*-factors are usually very close to the free electron *g*-value, with typical values in the range 1.99–2. The values of *g*_⊥_ and *g*_∥_ in Table 1 and fall in the normal range for Gd^3+^. In the case of **1**, **2**, and **3**, the *g* was modeled as isotropic because, when allowed to refine as axial, the values of *g*_∥_ and *g*_⊥_ were close and the standard deviations were significantly larger than the difference. **4** and **5** were modeled with anisotropic *g*-values. The direction of the *g*-anisotropy is opposite in these two compounds: **4** has *g*_⊥_ > *g*_∥_ and **5** the reverse. No explanation for this behavior presents itself.

*B*_2_^0^ is a measure of the axial crystal field. Here, we find the magnitude of *B*_2_^0^ follows the order opposite to the crystal field strength expected of the axial ligands. This observation is in line with already established results in the literature.^23^ The uncharged ligands in the plane of the bipyramid play only a minor role in defining the crystal field.

**Figure 6:**
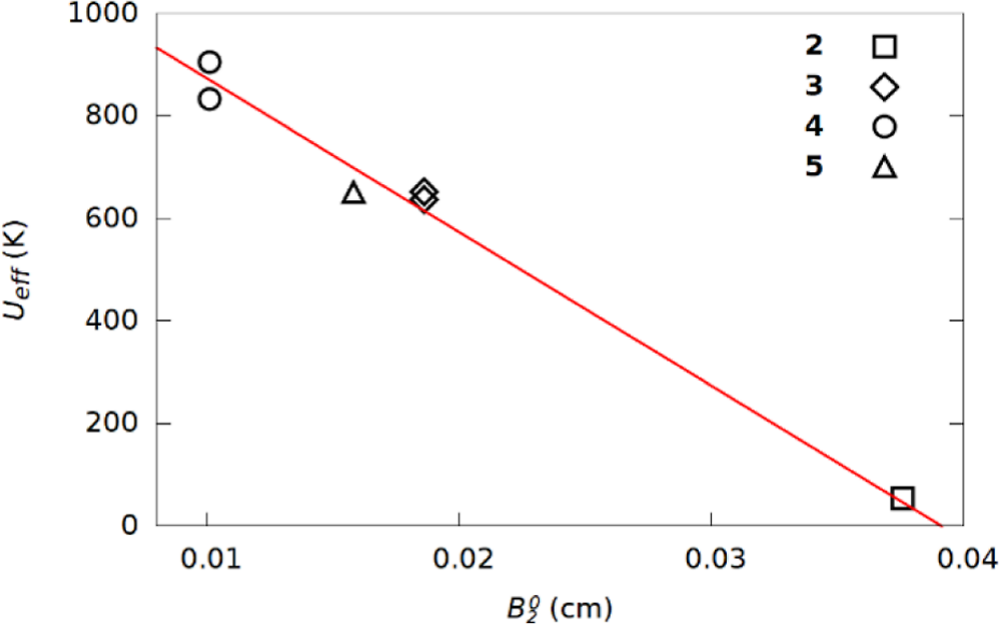
Comparison of the magnitude of *B*_2_^0^ and *U*_eff_ determined for the corresponding Dy^3+^ complex. [Dy(OPh)_2_Py_5_] is used as corresponding to [Gd(OArF_5_)_2_Py_5_] (**4**). The red line is a trendline to guide the eye. Two data points for *U*_eff_ are given where measurements of pure and doped Dy SMMs were reported.

The thermal energy barrier (*U*_eff_) of Dy^3+^ SMMs is always considered to be proportional to the axial crystal field. However, the axial crystal field has not been measured in most Dy^3+^ SMMs but rather calculated by CASSCF. There are significant exceptions.^26^ Here, we have measured *B*_2_^0^ directly in a series of Gd^3+^ compounds. **2**, **3**, and **5** all have Dy^3+^ analogues with published values of *U*_eff_.^14^ No Dy^3+^ analogues of **1** or **4** have been published, but a version of **4** with non-fluorinated phenoxide ligands is known with *U*_eff_ reported, though fluorinating the ligand could change the *U*_eff_ somewhat.^14,24^ Taking this series of compounds, we find that *B*_2_^0^ in the Gd^3+^ complexes has an inverse and linear correlation to the measured *U*_eff_ of the corresponding Dy^3+^ compounds (Figure 6). We propose that the inverse relationship is because ZFS in Gd^3+^ compounds arise from mixing of excited states.

The remaining axial ZFS parameters *B*_4_^0^ and *B*_6_^0^ have less influence on the spectra and are poorly defined. In particular, *B*_6_^0^ is of little importance and was so inadequately determined in the fits of **1**, **2**, and **3** that it was removed from the model.

The role of the  operator is to mix *m*_s_ states differ by Δ*m*_s_ = ±5: in the case of gadolinium that is mixing of the |±7/2> state with the |∓3/2> state and the intermixing of the |±5/2> states. *B*_6_^5^ is the only off-diagonal parameter allowed to be non-zero under *C*_5_ symmetry, so these states are therefore the only ones interacting at zero field. The compositions of the zero-field eigenstates derived from the ZFS parameters are given in Tables S6–S11.

Since *B*_6_^5^ is not allowed in *D*_5*h*_ symmetry, which is the idealized symmetry of the complexes other than **3**, we explored the correlation between the magnitude of *B*_6_^5^ and the deviation from this symmetry. In order to test this hypothesis, continuous shape measures (CShMs)^25^ were used as a parameter to describe the deviation of the first coordination sphere from pentagonal bipyramidal. To compare the *B*_6_^5^ parameters between the complexes, they were normalized by the overall splitting of the ^8^S_7/2_ state in zero-field, Δ*E* (final column in Table 1). At first glance there is no obvious connection between CShM and *B*_6_^5^ [we neglect compound **2** here because *B*_6_^5^ is ill-defined due to the *B*_2_^2^ term and two crystallographic sites]. The order is **1** < **5** < **3** < **4** for CShM, while it is **1** < **3** < **5** < **4** for *B*_6_^5^. Thus, the ordering fails with regards to **3** and **5**. Looking closer at **5**, it is found that despite it having a low CShM value, the X_1_-Gd-X_2_ angle for the axial ligands is the furthest from 180° of the five complexes. The deformation of the axiality seems therefore to influence the off-diagonal ZFS more than distortions to the equatorial ligands, possibly due to the higher charge.

We also considered the use of the *B*_2_^2^ parameter to fit the spectra. In one case (**2**), this was more effective to simulate the spectra than *B*_6_^5^. *B*_2_^2^ is not allowed if there is a fivefold rotation symmetry; it is a rhombic term. While we cannot draw strong conclusions from which of these two terms is used in which case, it is clear that the site symmetry in these SMMs as determined by EPR spectroscopy is never *D*_5*h*_. This matches the ChSM, based on the X-ray structures, which also shows a symmetry below *D*_5*h*_.

## Conclusions

Six crystalline powder samples of pentagonal bipyramidal complexes of pure Gd^3+^ and Gd^3+^ doped into Y^3+^ have been prepared and investigated with EPR at variable frequency. They were found to give well-resolved spectra.

The EPR spectra could be simulated by splitting of the ground state with a spin-Hamiltonian based on the restrictions of *D*_5*h*_ symmetry but some off-axis terms were needed in all cases. Some correlation between the CShM of the complex toward *D*_5*h*_ point group symmetry and the relative magnitude of the off-diagonal parameter of the ZFS was found. However, it was also found that distortions in the positions of the axial ligands have more impact than distortions of the ligands in the plane, possibly due to the larger influence of the axial ligands on the crystal field. Furthermore, the nature of the axial ligands is the determining factor for the magnitude of the *B*_6_^5^ parameter.

The magnitude of *B*_2_^0^ is inversely proportional to the crystal field strength expected for the axial ligands. We therefore investigated whether this correlates with the thermal energy barrier (*U*_eff_) for the Dy^3+^ analogues of these compounds which are SMMs. There is a good inverse correlation (Figure 6). The correlation is inverse because while the crystal field splitting in the Dy^3+^ SMMs is directly proportional to the *U*_eff_ in the Gd^3+^ complexes, the ZFS is due to mixing in of excited states into the ground state.

The energy barrier in lanthanide SMMs is often related to the crystal field splitting, and this has been regularly confirmed by high-level calculations.^14−16,18,26−29^ We have also reported a linear correlation between *U*_eff_ and *R* cos(π/(180 – θ)), where *R* = the Dy-axial ligand distance and θ is the angle at Dy between the axial ligands.^30^ The EPR data reported here are rare experimental confirmation that this is correct. In the future, we will investigate whether this correlation of *B*_2_^0^ for Gd^3+^ correlates with *U*_eff_ in other Dy^3+^ SMMs.

## Supporting Information Available

Crystallographic information, additional EPR spectra, and ground state compositions (PDF)

## References

[ref1] Abragam A.; Bleaney B. Electron Paramagnetic Resonance of Transition Ions, 1st ed.; Oxford : Clarendon press, 1970.

[ref2] Buckmaster H. A.; Shing Y. H. A Survey of the EPR Spectra of Gd3+ in Single Crystals. Phys. Status Solidi A 1972, 12, 325–361. 10.1002/pssa.2210120202.

[ref3] Buch C. D.; Kundu K.; Marbey J. J.; van Tol J.; Weihe H.; Hill S.; Piligkos S. Spin–Lattice Relaxation Decoherence Suppression in Vanishing Orbital Angular Momentum Qubits. J. Am. Chem. Soc. 2022, 144, 17597–17603. 10.1021/jacs.2c07057. 36106369

[ref4] López-Cabrelles J.; Escalera-Moreno L.; Hu Z.; Prima-García H.; Espallargas G. M.; Gaita-Ariño A.; Coronado E. Near Isotropic *D*_4*d*_ Spin Qubits as Nodes of a Gd(III)-Based Metal–Organic Framework. Inorg. Chem. 2021, 60, 8575–8580. 10.1021/acs.inorgchem.1c00504. 34096277 PMC8291595

[ref5] Luis F.; Alonso P. J.; Roubeau O.; Velasco V.; Zueco D.; Aguilà D.; Martínez J. I.; Barrios L. A.; Aromí G. A dissymmetric [Gd_2_] coordination molecular dimer hosting six addressable spin qubits. Commun. Chem. 2020, 3, 176 10.1038/s42004-020-00422-w. 36703386 PMC9814487

[ref6] Blagg R. J.; Ungur L.; Tuna F.; Speak J.; Comar P.; Collison D.; Wernsdorfer W.; McInnes E. J. L.; Chibotaru L. F.; Winpenny R. E. P. Magnetic Relaxation Pathways in Lanthanide Single-Molecule Magnets. Nat. Chem. 2013, 5, 673 10.1038/nchem.1707. 23881498

[ref7] Gatteschi D.; Sessoli R. Quantum Tunneling of Magnetization and Related Phenomena in Molecular Materials. Angew. Chem., Int. Ed. 2003, 42, 268–297. 10.1002/anie.200390099. 12548682

[ref8] Newman D. J. Origin of the Ground State Splitting of Gd3+ in Crystals. Chem. Phys. Lett. 1970, 6, 288–290. 10.1016/0009-2614(70)85077-1.

[ref9] Sørensen M. A.; Weihe H.; Vinum M. G.; Mortensen J. S.; Doerrer L. H.; Bendix J. Imposing High-Symmetry and Tuneable Geometry on Lanthanide Centres with Chelating Pt and Pd Metalloligands. Chem. Sci. 2017, 8, 3566–3575. 10.1039/C7SC00135E. 30155201 PMC6092721

[ref10] Senn F.; Helm L.; Borel A.; Daul C. A. Electronic Fine Structure Calculation of [Gd(DOTA)(H_2_O)]– Using LF-DFT: The Zero Field Splitting. C. R. Chim. 2012, 15, 250–254. 10.1016/j.crci.2011.10.008.

[ref11] Lasoroski A.; Vuilleumier R.; Pollet R. Vibrational Dynamics of Zero-Field-Splitting Hamiltonian in Gadolinium-Based MRI Contrast Agents from Ab Initio Molecular Dynamics. J. Chem. Phys. 2014, 141, 014201 10.1063/1.4885848. 25005282

[ref12] Ding Y.-S.; Chilton N. F.; Winpenny R. E. P.; Zheng Y.-Z. On Approaching the Limit of Molecular Magnetic Anisotropy: A Near-Perfect Pentagonal Bipyramidal Dysprosium(III) Single-Molecule Magnet. Angew. Chem., Int. Ed. 2016, 55, 16071–16074. 10.1002/anie.201609685. 27874236

[ref13] Ding Y.-S.; Yu K.-X.; Reta D.; Ortu F.; Winpenny R. E. P.; Zheng Y.-Z.; Chilton N. F. Field- and Temperature-Dependent Quantum Tunnelling of the Magnetisation in a Large Barrier Single-Molecule Magnet. Nat. Commun. 2018, 9, 3134 10.1038/s41467-018-05587-6. 30087339 PMC6081483

[ref14] Ding Y.-S.; Han T.; Zhai Y.-Q.; Reta D.; Chilton N. F.; Winpenny R. E. P.; Zheng Y.-Z. A Study of Magnetic Relaxation in Dysprosium(III) Single-Molecule Magnets. Chem.—Eur. J. 2020, 26, 5893–5902. 10.1002/chem.202000646. 32073707

[ref15] Gupta S. K.; Rajeshkumar T.; Rajaraman G.; Murugavel R. An Air-Stable Dy(III) Single-Ion Magnet with High Anisotropy Barrier and Blocking Temperature. Chem. Sci. 2016, 7, 5181–5191. 10.1039/C6SC00279J. 30155168 PMC6020529

[ref16] Gupta S. K.; Rajeshkumar T.; Rajaraman G.; Murugavel R. An Unprecedented Zero Field Neodymium(III) Single-Ion Magnet Based on a Phosphonic Diamide. Chem. Commun. 2016, 52, 7168–7171. 10.1039/C6CC03066A. 27173026

[ref17] Gupta S. K.; Murugavel R. Enriching Lanthanide Single-Ion Magnetism Through Symmetry and Axiality. Chem. Commun. 2018, 54, 3685–3696. 10.1039/C7CC09956H. 29564454

[ref18] Gupta S. K.; Rajeshkumar T.; Rajaraman G.; Murugavel R. Is a Strong Axial Crystal-Field the Only Essential Condition for a Large Magnetic Anisotropy Barrier? The Case of Non-Kramers Ho(III) versus Tb(III). Dalton Trans. 2018, 47, 357–366. 10.1039/C7DT04020B. 29215670

[ref19] Stevens K. W. H. Matrix Elements and Operator Equivalents Connected with the Magnetic Properties of Rare Earth Ions. Proc. Phys. Soc., London, Sect. A 1952, 65, 209 10.1088/0370-1298/65/3/308.

[ref20] Jacobsen C. J. H.; Pedersen E.; Villadsen J.; Weihe H. ESR Characterization of Trans-Diacidatotetrakis(Pyridine)Vanadium and -Manganese Trans-VII(Py)_4_X_2_ and Trans-MnII(Py)_4_X_2_ (X = NCS, Cl, Br, I; Py = Pyridine). Inorg. Chem. 1993, 32, 1216–1221. 10.1021/ic00059a031.

[ref21] Husein Mor H.; Weihe H.; Bendix J. Fitting of EPR Spectra: The Importance of a Flexible Bandwidth. J. Magn. Reson. 2010, 207, 283–286. 10.1016/j.jmr.2010.09.011. 20937565

[ref22] Shannon R. D. Revised Effective Ionic Radii and Systematic Studies of Interatomic Distances in Halides and Chalcogenides. Acta Crystallogr., Sect. A: Cryst. Phys., Diffr., Theor. Gen. Crystallogr. 1976, 32, 751–767. 10.1107/S0567739476001551.

[ref23] Levin L. I.; Gorlov A. D. Gd3+ Crystal-Field Effects in Low-Symmetric Centres. J. Phys.: Condens. Matter 1992, 4, 1981–1992. 10.1088/0953-8984/4/8/013.

[ref24] Ma Y.; Zhai Y.-Q.; Luo Q.-C.; Ding Y.-S.; Zheng Y.-Z. Ligand Fluorination to Mitigate the Raman Relaxation of DyIII Single-Molecule Magnets: A Combined Terahertz, Far-IR and Vibronic Barrier Model Study. Angew. Chem., Int. Ed. 2022, 61, e202206022 10.1002/anie.202206022. 35543224

[ref25] Alvarez S.; Alemany P.; Casanova D.; Cirera J.; Llunell M.; Avnir D. Shape Maps and Polyhedral Interconversion Paths in Transition Metal Chemistry. Coord. Chem. Rev. 2005, 249, 1693–1708. 10.1016/j.ccr.2005.03.031.

[ref26] Norel L.; Darago L. E.; Le Guennic B.; Chakarawet K.; Gonzalez M. I.; Olshansky J. H.; Rigaut S.; Long J. R. A Terminal Fluoride Ligand Generates Axial Magnetic Anisotropy in Dysprosium Complexes. Angew. Chem., Int. Ed. 2018, 57, 1933–1938. 10.1002/anie.201712139. 29285845

[ref27] Goodwin C. A. P.; Ortu F.; Reta D.; Chilton N. F.; Mills D. P. Molecular Magnetic Hysteresis at 60 Kelvin in Dysprosocenium. Nature 2017, 548, 439–442. 10.1038/nature23447. 28836589

[ref28] Randall McClain K.; Gould C. A.; Chakarawet K.; Teat S.; Groshens T. J.; Long J. R.; Harvey B. G. High-Temperature Magnetic Blocking and Magneto-Structural Correlations in a Series of Dysprosium(III) Metallocenium Single-Molecule Magnets. Chem. Sci. 2018, 9, 8492–8503. 10.1039/C8SC03907K. 30568773 PMC6256727

[ref29] Guo F.-S.; Day B. M.; Chen Y.-C.; Tong M.-L.; Mansikkamäki A.; Layfield R. A. Magnetic Hysteresis up to 80 Kelvin in a Dysprosium Metallocene Single-Molecule Magnet. Science 2018, 362, 1400–1403. 10.1126/science.aav0652. 30337456

[ref30] Ding Y.-S.; Winpenny R. E. P.; Zheng Y.-Z. 3d and 4f-based single molecule magnets. In Comprehensive Coordination Chemistry III; Constable E. C., Parkin G., Que L. Jr., Eds., 2021; Vol. 9, pp 595–619.

