# Supplementary material for: Electron Paramagnetic Resonance Spectra of Pentagonal Bipyramidal Gadolinium Complexes
Source: Inorg Chem. 2023 May 12;62(21):8435–41. doi: 10.1021/acs.inorgchem.3c01227 (PMC10230502; doi:10.1021/acs.inorgchem.3c01227)
Supplement: Supplementary file 1 — ic3c01227_si_001.pdf [file ic3c01227_si_001.pdf]

Supporting information

Electron Paramagnetic Resonance Spectra of Pentagonal Bipyramidal Gadolinium Complexes

Jonatan B. Petersen,[1] You-Song Ding,[1,2] Sandeep K. Gupta,[3] Aditya Borah,[3] Eric J. L. McInnes,[1] Yan-Zhen Zheng,[2] Ramaswamy Murugavel,[3] and Richard E. P. Winpenny[1]*

[1] Department of Chemistry, School of Natural Science, The University of Manchester, Oxford Road, Manchester M13 9PL, United Kingdom

[2]Frontier Institute of Science and Technology (FIST), Xi'an Jiaotong University, 99 Yanxiang Road, Xi'an 710049, China

[3] Department of Chemistry, Indian Institute of Technology Bombay, Mumbai-400076, India

Crystallography

Table S1*. Crystallographic and refinement parameters for compounds* ***1*** *-* ***5***

	$[YCl_2(Py)_5]$ BPh_4·THF (**1**)	$YCl_2(THF)_5$ BPh_4· 0.5THF (**2**)	$[Y(O^tBu)Cl(THF)_5]BPh_4$· 2THF (**3**)
Reference	This work	This work	1,2
Sum formula	$C_{49}H_{45}BCl_2N_5Y$	$C_{46}H_{64}BC_2O_{5.5}Y$	$YC_{56}H_{85}BClO_8$
Formula weight (g/mol)	874.5	875.59	1021.4
Crystal system	Orthorhombic	Triclinic	Monoclinic
Space group	$Pca2_1$	$P\bar{1}$	$P2_1/n$
T (K)	150	150	100
a (Å)	19.8587(16)	12.5396(6)	13.6356(17)
b (Å)	12.5584(10)	12.7786(6)	12.4340(15)
c (Å)	18.0227(17)	29.3605(15)	32.618(4)
α (°)	90	79.595(4)	90
β (°)	90	79.953(4	90.434(2)
γ (°)	90	82.294(4)	90
V (Å^3)	4494.7(7)	4528.2(4)	5530.0(12)
Z	4	4	4
ρ_{calc} (g cm^{-3})	1.292	1.284	1.227
μ (mm^{-1})	1.455	1.450	1.153
F_{000}	1808	1848	2184
θ (°)	3.4450 -20.4830	3.150 – 27.266	2.22 – 23.43
Goodness of fit	1.055	1.014	1.049
R_1 (%)	8.06	6.01	5.85
wR_2 (%)	8.91	12.51	15.78

Table S1 continued**. Crystallographic and refinement parameters for compounds **1** - **5

	$[Y(OArF_5)_2(Py)_5]$ $B(ArF_5)_4\cdot 0.5C_6H_{14}$ (**4**)	$[Y(^tBuPO(NH^iPr)_2)_2$ $(H_2O)_5][I]_3\cdot H_2O$ $\cdot 2(^tBuPO(NH^iPr)_2$ (**5**)	$[Gd(^tBuPO(NH^iPr)_2)_2$ $(H_2O)_5][I]_3\cdot H_2O$ $\cdot 2(^tBuPO(NH^iPr)_2$ (**5b**)
Reference	This work	3	This work
Sum formula	$YC_{64}H_{32}BF_{30}N_5O_2$	$YC_{40}H_{112}I_3N_8O_{10}P_4$	$GdC_{40}H_{112}I_3N_8O_{10}P_4$
Formula weight (g/mol)	1572.7	1458.9	1527.20
Crystal system	Triclinic	Triclinic	Triclinic
Space group	$P\bar{1}$	$P\bar{1}$	$P\bar{1}$
T (K)	150	120	120
a (Å)	11.9649(8)	13.7506(13)	13.7931(11)
b (Å)	14.5150(10)	14.727(2)	14.7302(9)
c (Å)	19.4283(16)	20.641(2)	20.712(2)
α (°)	73.514(7)	92.293(14)	92.658(17)
β (°)	77.693(6)	108.245(10)	108.510(13)
γ (°)	79.769(6)	114.902(8)	114.902(5)
V (Å^3)	3136.3(4)	3528.3(7)	3539.3(6)
Z	2	2	2
ρ_{calc} (g cm^{-3})	1.665	1.373	1.433
μ (mm^{-1})	1.069	2.275	2.381
F_{000}	1562	1492	1542
θ (°)	2.712-28.961	2.205-25.250	2.659-25.250
Goodness of fit	1.064	1.060	1.080
R_1 (%)	9.36	3.36	3.45
wR_2 (%)	22.89	7.62	8.60

Table S2. Selected bond lengths (Å) and angles (°)

	**1**[a]	**2**[b]	**3**[c]	**4**[a]	**5**[c]
Y-X bonds (axial)					
Y1-Cl1	2.574(3)	2.5653(9)	2.6608(11)		
Y1-Cl2	2.576(3)	2.5675(9)			
Y2-Cl3		2.5842(9)			
Y2-Cl4		2.5649(10)			
Y1-O1			2.042(3)	2.143(4)	2.206(2)
Y1-O2				2.150(4)	2.201(2)
Y – E bonds (equatorial)					
Y1-E1	2.514(7)	2.394(2)		2.522(5)	
Y1-E2	2.536(8)	2.387(2)	2.421(3)	2.506(5)	
Y1-E3	2.506(9)	2.382(2)	2.414(3)	2.500(5)	
Y1-E4	2.509(6)	2.382(2)	2.418(3)	2.523(5)	
Y1-E5	2.516(8)	2.396(2)	2.412(3)	2.512(6)	2.3478(14)
Y1-O6			2.390(3)		2.3421(13)
Y1-O7					2.3428(15)
Y1-O8					2.355(2)
Y1-O9					2.3465(18)
Y2-O6		2.391(2)			
Y2-O7		2.369(2)			
Y2-O8		2.411(2)			
Y2-O9		2.383(2)			
Y2-O10		2.368(2)			
X-Y-X angles (axial)					
Cl1-Y1-Cl2	177.42(8)	176.43(3)			
Cl3-Y2-Cl4		179.61(3)			
O1-Y1-Cl1			178.72(8)		
O1-Y1-O2				178.59(17)	175.02(8)
Angles at Y involving equatorial atoms					
Range nearest E-Y-E	69.7(2) – 74.1(3)	71.47(8) – 72.46(8)	71.43(10) – 72.82(10)	70.94(16) – 73.67(18)	70.57(7) – 73.37(9)
Range Cl-Y-E	88.0(2) – 93.0(2)	86.36(6) – 94.68(6)	83.91(7) – 89.16(7)		
Range O_{ax}-Y-E_{eq}			90.20(10) – 95.59(10)	84.06(16) – 97.33(17)	86.03(8) – 94.51(8)

a. E = N
b. E = O; two molecules in the asymmetric unit
c. E = O

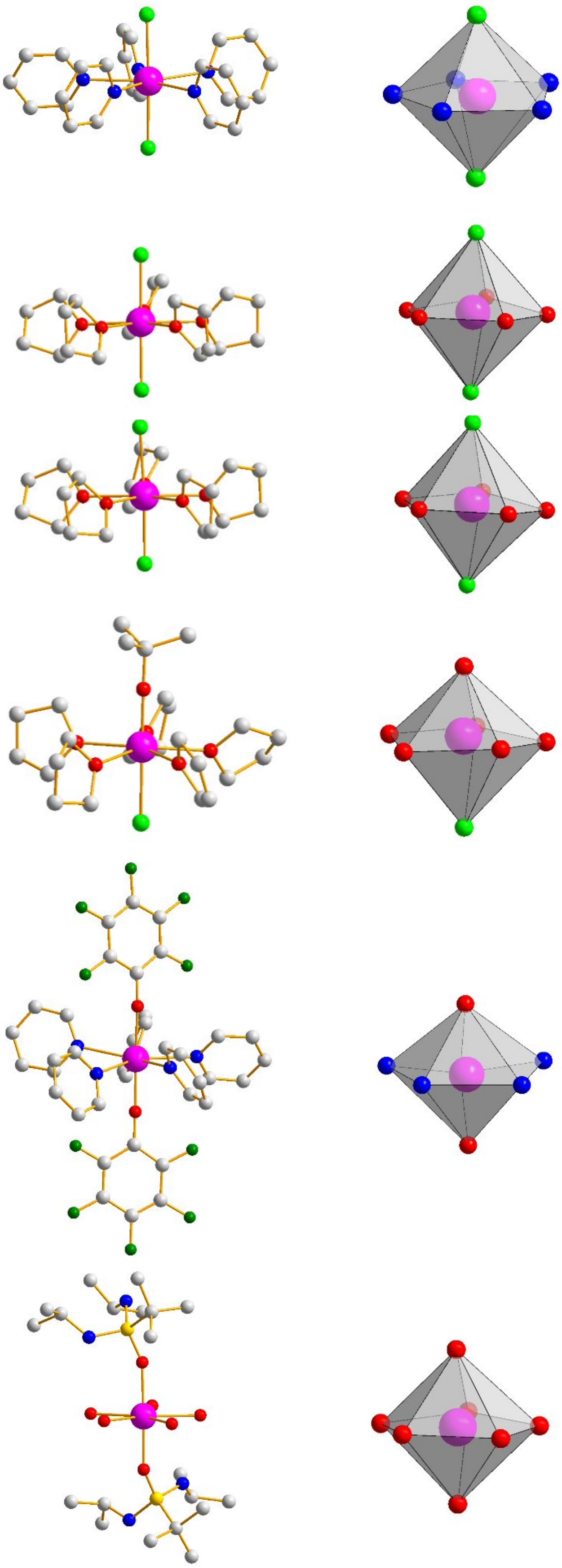

$[GdCl_2(Py)_5]BPh_4\cdot THF$ (**1**)

$2[GdCl_2(THF)_5]2BPh_4\cdot THF$ (**2**)

Site 1

Site 2

$[Y(O^tBu)Cl(THF)_5]BPh_4\cdot 2THF$ (**3**)

$[Y(OArF_5)_2(Py)_5]B(ArF_5)_4\cdot 0.5C_6H_{14}$(**4**)

$[Y(^tBuPO(NH^iPr)_2)_2(H_2O)_5][I]_3\cdot H_2O\cdot 2(^tBuPO(NH^iPr)_2$ (**5**)

Figure S1. *Solid state structures of the 5 complexes, excluding counterions and solvent. Colour code: Gd/Y=magenta, P=yellow, Cl=light green, F=dark green, O=red, N=Blue and C=grey. Hydrogen is not shown*

EPR spectroscopy

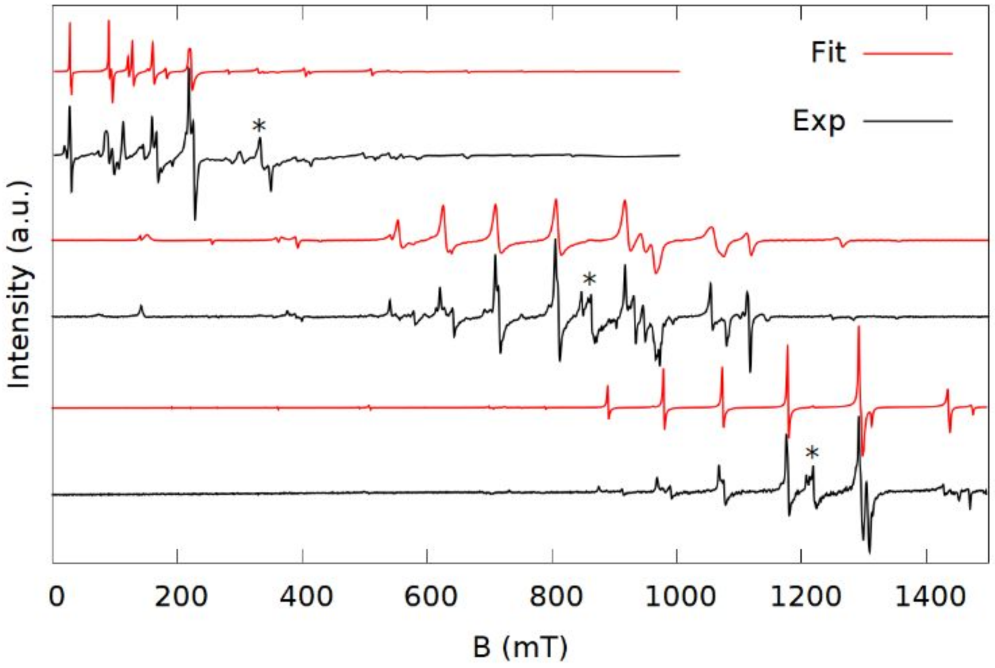

***Figure S2**. Spectra (black) and simulations (red) of **2** at X (top), K (middle) and Q-band (bottom). Fitted with the spin Hamiltonian of equation 1 without the B_2^2 term (See manuscript for discussion).*

***Table S3**. Best fit spin Hamiltonian parameters for **2** from fit of EPR spectra using spin Hamiltonian containing B_6^5. Numbers in parenthesis are estimated standard deviations of the last digit.*

	$g_\perp$	$g_\parallel$	B_2^0 10^{-2} cm^{-1}	B_4^0 10^{-5} cm^{-1}	B_6^5 10^{-5} cm^{-1}
**2**	1.9910(2)	-	3.761(1)	-0.921.2(41)	5.0(4)

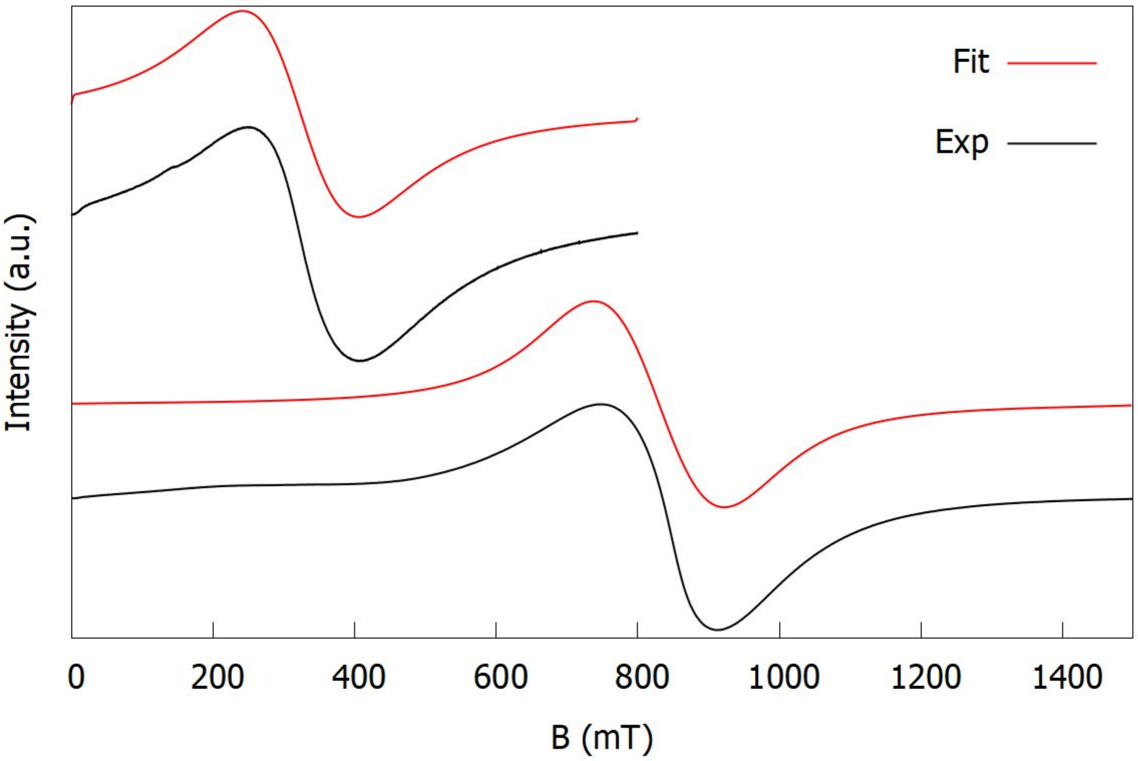

Figure S3. *Spectra (black) and simulations (red) of neat $GdCl_2(THF)_5$ at X (top) and K (bottom). Simulated with S=1/2.*

Table S4. *Best fit spin Hamiltonian parameters for* ***5b*** *from fit of X- and Q-band EPR spectra. Numbers in parenthesis are estimated standard deviations of the last digit.*

**5**	$g_{\perp}$	$g_{\parallel}$	B_2^0 10^{-2} cm^{-1}	B_4^0 10^{-5} cm^{-1}	B_6^0 10^{-7} cm^{-1}	B_6^5 10^{-5} cm^{-1}	ΔE (cm^{-1})	$B_6^5/\Delta E$ (10^{-5})
X-band	1.988(4)	1.997(7)	1.47(2)	-0.3(2)	-4(1)	7.2(7)	0.527	14
Q-band*	1.989(3)*	1.995(6)*	1.6(1)	-3(1)	-4(5)	6(2)	0.58	10

*not field corrected

Parameter conversion

In order to convert the P_k^q parameters obtained via the multi purpose EPR fitting program to the B_k^q parameters as originally defined by Stevens we used the relation.[4,5]

$$B_{q,Wybourne}^{k} = \frac{P_k^q}{2^k}\sqrt{\frac{(2S+k+1)!}{(2S-k)!}}$$

Which results in parameters normalised in Wybourne's notation. These were then divided by the ratios listed in Table S4 to convert them to Stevens parameters.[6]

$$B_{k,Stevens}^{q} = \frac{B_{q,Wybourne}^{k}}{\lambda_{k,q}}$$

Table S5. *Conversion factors between Stevens and Wybourne normalised parameters.*

k	q	$\lambda_{k,q}$
2	0	2
2	2	$\frac{2}{\sqrt{6}}$
4	0	8
6	0	16
6	5	$\frac{-8}{3\sqrt{77}}$

Ground state compositions

Table S6*. Composition of the eigenstates of* ***1*** *in the* m_S *state basis.*

m_S\E(cm^{-1})	0.000	0.000	0.227	0.227	0.671	0.671	1.308	1.308
-3.5			0.00016				99.99984	
-2.5						100		
-1.5				99.99984				0.00016
-0.5	100							
0.5		100						
1.5			99.99984				0.00016	
2.5					100			
3.5				0.00016				99.99984

Table S7*. Composition of the eigenstates of* ***2*** *in the* m_S *state basis.*

m_S\E(cm^{-1})	0.000	0.000	0.234	0.234	0.693	0.693	1.356	1.356
-3.5			0.0153				99.9847	
-2.5						100		
-1.5				99.9847				0.0153
-0.5	100							
0.5		100						
1.5			99.9847				0.0153	
2.5					100			
3.5				0.0153				99.9847

Table S8*. Composition of the eigenstates of* ***2*** *in the* m_S *state basis using alternative spin-hamiltonian.*

m_S\E(cm^{-1})	0.000	0.000	0.234	0.234	0.691	0.691	1.357	1.357
-3.5		0.00001		0.00554			99.99444	
-2.5	0.03139		0.00026		99.96833	0.00001		
-1.5	0.00005	0.36631		99.62805		0.00003	0.00556	
-0.5	99.58996	0.01228	0.36614		0.03163			
0.5	0.01228	99.58996		0.36614		0.03163		
1.5	0.36631	0.00005	99.62805		0.00003			0.00556

2.5		0.03139		0.00026	0.00001	99.96833		
3.5	0.00001		0.00554					99.99444

***Table S9**. Composition of the eigenstates of **3** in the m_S state basis.*

m_S\E(cm^{-1})	0.000	0.000	0.123	0.123	0.356	0.356	0.673	0.673
-3.5			0.0071				99.9929	
-2.5						100		
-1.5				99.9929				0.0071
-0.5	100							
0.5		100						
1.5			99.9929				0.0071	
2.5					100			
3.5				0.0071				99.9929

***Table S10**. Composition of the eigenstates of **4** in the m_S state basis.*

m_S\E(cm^{-1})	0.0000	0.0000	0.0551	0.0551	0.2078	0.2078	0.3611	0.3611
-3.5			0.4964				99.5036	
-2.5					100			
-1.5				99.5036				0.4964
-0.5	100							
0.5		100						
1.5			99.5036				0.4964	
2.5						100		
3.5				0.4964				99.5036

***Table S2**. Composition of the eigenstates of **5b** in the m_S state basis.*

m_S\E(cm^{-1})	0.000	0.000	0.106	0.106	0.323	0.323	0.568	0.568
-3.5			0.1624				99.8376	
-2.5						100		
-1.5				99.8376				0.1624
-0.5		100						
0.5	100							
1.5			99.8376				0.1624	
2.5					100			

3.5				0.1624				99.8376

References

(1) Ding, Y.-S.; Yu, K.-X.; Reta, D.; Ortu, F.; Winpenny, R. E. P.; Zheng, Y.-Z.; Chilton, N. F. Field- and Temperature-Dependent Quantum Tunnelling of the Magnetisation in a Large Barrier Single-Molecule Magnet. *Nat. Commun.* **2018**, *9* (1), 3134. https://doi.org/10.1038/s41467-018-05587-6.

(2) Evans, W. J.; Olofson, J. M.; Ziller, J. W. Synthesis and Structure of the Cationic Tert-Butoxide Complexes Y3(OR)7Cl(THF)3+, Y2(OR)4Cl(THF)4+, and Y(OR)Cl(THF)5+: Representatives of a New Class of Yttrium Alkoxides. *J. Am. Chem. Soc.* **1990**, *112* (6), 2308–2314. https://doi.org/10.1021/ja00162a035.

(3) Gupta, S. K.; Rajeshkumar, T.; Rajaraman, G.; Murugavel, R. An Air-Stable Dy(Iii) Single-Ion Magnet with High Anisotropy Barrier and Blocking Temperature. *Chem. Sci.* **2016**, *7* (8), 5181–5191. https://doi.org/10.1039/C6SC00279J.

(4) Rudowicz, C.; Chung, C. Y. The Generalization of the Extended Stevens Operators to Higher Ranks and Spins, and a Systematic Review of the Tables of the Tensor Operators and Their Matrix Elements. *J. Phys. Condens. Matter* **2004**, *16* (32), 5825–5847. https://doi.org/10.1088/0953-8984/16/32/018.

(5) D Smith; J H M Thornley. The Use of `operator Equivalents'. *Proc. Phys. Soc.* **1966**, *89* (4), 779. https://doi.org/10.1088/0370-1328/89/4/301.

(6) Newman, D. J.; Betty, N. G. Empirical Crystal Fields. In *Crystal Field Handbook*; Cambridge University Press, 2000. https://doi.org/10.1017/CBO9780511524295.
